# Workplace violence and burnout among Chinese nurses during the COVID-19 pandemic: does the sense of coherence mediate the relationship?

**DOI:** 10.1186/s12888-023-05060-9

**Published:** 2023-08-08

**Authors:** Yeping Fei, Silan Yang, Zhihong Zhu, Mengmeng Lv, Yan Yin, Man Zuo, Yiping Chen, Han Sheng, Shenya Zhang, Mingmin Zhang

**Affiliations:** 1 The First Hospital of Jiaxing, Affiliated Hospital of Jiaxing University, No. 1882, Zhonghuan South Road, Jiaxing City, 314000 Zhejiang Province China; 2 Alice Lee Centre for Nursing Studies, Yong Loo Lin School of Medicine, National University of Singapore, Singapore, Singapore; 3 The First Affiliated Hospital of University of South China, Hengyang, Hunan China; 4 Heyuan Hospital of Guangdong Provincial People’s Hospital, Heyuan, Guangdong China; 5 Department of Gastroenterology, Shenzhen Hospital, Southern Medical University, Shenzhen, Guangdong China

**Keywords:** Burnout, Workplace violence, Nurses, Sense of coherence, Mediation

## Abstract

**Background:**

Workplace violence has always been a critical issue worldwide before and after the COVID-19 pandemic, which can lead to burnout and turnover. In addition, the burnout and mental stress of nurses during the COVID-19 period have been widely described. To our knowledge, no studies have examined the mediating effect of the sense of coherence on the relationship between workplace violence and burnout among Chinese nurses over time. Therefore, this study aimed to explore the relationship between workplace violence and burnout among Chinese nurses and how the sense of coherence mediates the association.

**Methods:**

Using a convenience sampling method, 1190 nurses from 4 tertiary grade-A comprehensive hospitals were investigated between September 2021 and December 2021 in 3 provinces of China. The Workplace Violence Scale, Burnout Inventory, and Sense of Coherence scale were used to collect data. Data analysis was conducted using descriptive statistics, Pearson correlation, and regression analysis to estimate direct and indirect effects using bootstrap analysis.

**Results:**

The mean total scores for workplace violence and burnout were 1.67 ± 1.08 and 47.36 ± 18.39, respectively. Workplace violence was significantly negatively correlated with the sense of coherence (*r* = -0.25) and positively correlated with burnout (*r* = 0.27). Additionally, a higher level of workplace violence was associated with higher burnout (*β* = 1.13, 95% CI: 0.68 ~ 1.56). A higher sense of coherence was also associated with lower burnout (*β* = -0.98, 95% CI: -1.03 ~ -0.92). Workplace violence showed an effect on burnout through a sense of coherence. The direct, indirect and total effects were 1.13, 1.88 and 3.01, respectively. The mediating effect of the sense of coherence accounted for 62.45% of the relationship between workplace violence and burnout.

**Conclusion:**

We found that the sense of coherence mediated most workplace violence on burnout. It is imperative for hospital managers to improve nurses’ sense of coherence to reduce the occurrence of burnout during COVID-19. Future intervention studies should be designed to strengthen nurses’ sense of coherence.

**Supplementary Information:**

The online version contains supplementary material available at 10.1186/s12888-023-05060-9.

## Background

With the enhancement and improvement of people’s health awareness, patients’ demands for hospital nursing have also increased [1]. Nurses play a crucial role in health care. Due to multiple demands, high workload, and shift working hours, nurses have a high risk of psychological distress. For nurses, the pressure of a professional and social life along with occupational hazards leads to increased physical and mental fatigue as well as burnout [1]. Burnout influenced nurses’ turnover [2] and intention to leave [3] before and during the COVID-19 pandemic. Similarly, many psychosocial characteristics of the Chinese medical environment, such as workplace violence in clinical practice can also influence burnout [4, 5].

Workplace violence refers to incidents in which staff are abused, threatened, or assaulted in circumstances related to their work, including commuting to and from work, that involve an explicit or implicit challenge to their safety [6]. Most workplace violence is perpetrated by patients and visitors [7]. Workplace violence in health care is classified as physical and psychological violence (verbal abuse, threats, bullying, and sexual/racial harassment) [7]. Physical violence refers to the act of physically attacking others or groups and causing physical, sexual and spiritual harm, including beating, slapping, throwing, pushing, and biting [8]. Psychological violence refers to the intentional use of authority in personal threats against others or groups that causes harm to the body, spirit, soul, morality or social development, including verbal abuse, bullying/intimidation, harassment and threats [9]. The incidence of workplace violence for nurses remains high [8]. It has been a serious public problem in China before and during the COVID-19 pandemic as a negative work event [5, 10].Workplace violence has multiple effects on nurses’ bodies and minds. A large body of studies have suggested that exposure to workplace violence directly affects nurses’ job satisfaction, performance and burnout [11, 12]. In addition, there is a significant positive correlation between workplace violence and burnout in nurses [13]. The harm caused by workplace violence cannot be ignored.

Burnout refers to a psychological syndrome of emotional exhaustion, depersonalization and reduced sense of personal accomplishment [14], which can have negative consequences for the organizational health of the institution and the mental and physical health of nurses [15]. Before and during the pandemic, the levels of burnout among nurses were high [10, 16]. Burnout was found to be prevalent in 54.19% of 1,264 nurses during the COVID-19 pandemic [17]. Previous researchers have shown that a high level of nurses’ burnout could lead to high psychological distress, decreased job performance, and increased intention to leave [6]. The occupational stability and health problems of nurses have been emphasized by many nursing managers, and many scholars have tried to reduce the professional pressure on nurses from different perspectives such as resilience and capacity for mentalizing [18, 19]. In addition, sense of coherence (SOC) plays an important role in burnout [20] and is the main construct of the salutogenic model in coping with stress [21] which explores the impact of stress on human health through SOC and clarifies how to promote health and maintain a positive state for individuals facing stress [22].

SOC manifests as people’s readiness and willingness to utilize the resources at their disposal to appraise, understand, and make sense of their complex reality and environment and to choose appropriate strategies to deal with stressors and anxiety in spite of adversity [20, 23]. It expresses the degree to which a person has a diffuse, dynamic but lasting sense that stimuli are internal or external and that stressors are understandable (i.e., predictable, structured and explicable), manageable (i.e., there are resources available to meet the requirements of these stimuli) and meaningful (i.e., the requirements are challenges that are worth committing to and addressing) [24]. A previous study found that SOC played a mediating role between psychological load and burnout and affected Polish nurses’ burnout [25]. In addition, a study showed that SOC acted as a mediator and not as a moderator of relationships between exposure to violence and psychological, psychosomatic and cognitive stress reactions among Danish employees [26]. Similarly, a recent study found that an additional 8.3% of burnout was explained by the SOC among Greek nurses [24], and some studies have confirmed that SOC is negatively associated with burnout [27, 28]. Therefore, it can be assumed that nurses with a high SOC can better identify the nature of the stressors they face and select appropriate coping resources for these stressors. In summary, SOC may play a mediating role between workplace violence and burnout among Chinese nurses.

Most previous studies have examined workplace violence and burnout. However, to the best of our knowledge, there is little available information on the mediating role of SOC in the relationship between workplace violence and burnout among Chinese nurses during the COVID-19 pandemic. It is impossible to eliminate the occurrence of workplace violence toward nurses in clinical practice, but it can be changed by appropriate interventions for SOC [29].We aimed to explore the association between workplace violence and burnout among Chinese nurses and how SOC mediates this association based on a path analysis.

**Fig. 1:**
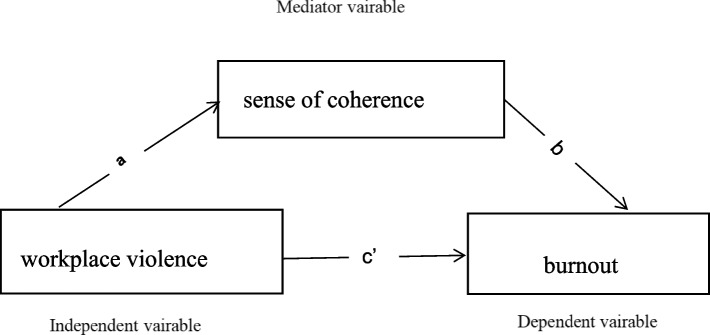
Hypothesized mediating model of workplace violence, sense of coherence and burnout. Note: **a** direct effect of workplace violence on sense of coherence; **b** direct effect of sense of coherence on burnout; **c** direct effect of workplace violence on burnout

To sum up, we propose a model of the impact of workplace violence on burnout that includes SOC as a potential mediator based on Jiang Qianjin’s stress system model [30]. The model suggests that there are many intermediary variables(cognitive evaluation, social support, personality and other psychological factors) between stressors and stress reactions [30]. We hypothesized that SOC (psychological factors) is a mediator of the relationship between workplace violence (stressors) and burnout (stress reactions) after controlling context variables (e.g., demographics) according to this model. Figure 1 showed a theoretical hypothesis model. We propose the following hypotheses: (1) workplace violence is negatively related to SOC; (2) workplace violence is positively related to burnout; (3) SOC is negatively related to burnout; and (4) SOC mediates the relationship between workplace violence and burnout.

## Methods

### Study population and procedure

This study followed the Strengthening the Reporting of Observational Studies in Epidemiology (STROBE) checklist for cross-sectional studies, developed by the EQUATOR Network (Enhancing the Quality and Transparency Of health Research) (Additional file 1: Appendix 1). A cross-sectional survey was conducted among nurses working in 4 tertiary grade-A comprehensive hospitals in Zhejiang, Hunan, and Guangdong provinces between September 2021 and December 2021. A convenience sampling method was used to collect the data because a face-to-face interview was limited due to COVID-19 [31] and busy work. A self-reported questionnaire was created using “Wenjuan Xing” (https://www.wjx.cn/), and the link to the online questionnaire was shared on major social media platforms (WeChat). The sample size was calculated by applying a single population proportion formula of qualitative variable data: *n* = (Z_1-α/2_)^2^p(1-p)/δ^2^. Because the total population proportion was not known, 50% was taken as the sample proportion of workplace violence [32], with a marginal error of 5, 95% CI (α = 0.05), *n* = 1.96^2^ × 0.5(1–0.5)/0.05^2^ = 384.16 ≈ 384. Considering a nonresponse rate of 20% [33],the final sample size was 835 nurses [384 × 2 × (1 + 0.2)].

#### Ethical approval and consent to participate

The study was approved by the ethics committee named the Medical Ethics Committee of Jiaxing First Hospital (LS2021-KY-413). Informed consent was placed on the homepage of the questionnaire, and the subjects clicked "informed consent" before entering the response interface. All information was only available to the research members, and the exported data were encrypted and saved. Before data collection, institutional consent was obtained after the purpose of the research was presented to the head of each participating hospital’s nursing department. The aim of the research was explained to the participants, and they were asked to fill out the consent forms online. All participants gave informed consent to the researchers before the survey, and the participants’ personal information was kept confidential.

#### Participants

The eligibility criteria were as follows: (1) direct contact with patients or their family members and (2) a nurse practice certificate. The exclusion criteria were as follows: (1) a family history of mental disease and (2) other violence (intimate partner violence, colleague violence).

A total of 1190 nurses participated in the present survey. After excluding questionnaires with missing data, 1105 nurses were included for analysis. The effective response rate in the current study was 92.85%.

#### Data quality control

The questionnaire was created with “Wenjuan Xing” (https://www.wjx.cn/), a platform of electronic questionnaires. We sent a web page of the questionnaire to participants’ mobile phones using a social media app (WeChat) [34]. For questionnaire quality control, (a) the questionnaire options were checked before submission; (b) each IP address was allowed to answer only once; (c) we set a reasonable jump to save nurses’ time and reduce invalid or incorrect information. In addition, questionnaires submitted in less than 3 min or more than 20 min were invalid.

#### Measures

##### Demographic characteristics questionnaire

Nurses’ demographic information was collected by a general information questionnaire designed for the research group according to the research purpose and rigorous literature review (Additional file 2: Appendix 2).

##### Workplace violence scale

Workplace violence was measured by the Workplace Violence Scale developed by the International Labour Office, the International Council of Nurses, the World Health Organization and the Public Services International Joint Program in 2003 [7]. This questionnaire is used to investigate the frequency of workplace violence experienced by research subjects in the past 12 months. It includes 9 items on physical violence and nonphysical violence. Each item is scored on a 4-point scale reflecting the frequency of respondents’ exposure to workplace violence in the past year (0 = never, 1 = once, 2 = twice, 3 = three or more times). The total possible times range from 0 to 27, and Cronbach’s α is 0.87 [11]. In the present study, the Cronbach’s α for the workplace violence scale was 0.85 (Additional file 2: Appendix 2).

##### Burnout inventory

Burnout was measured by the Maslach Burnout Inventory [35], which was revised and translated into Chinese in 2002 by Professor Peng, and has been widely used [36].The scale contains 3 dimensions (emotional exhaustion, depersonalization and personal accomplishment) and 22 items. Each item is rated on a 7-point Likert-type scale (from 0 = never to 6 = a few times a week), and items 4, 7, 9, 12, 17, 18, 19, and 21 are scored in reverse. The total score ranges from 0 to 132, and higher summed scores indicate higher burnout. The Cronbach’s α for the emotional exhaustion, depersonalization and personal accomplishment dimensions was 0.88, 0.83, and 0.82, respectively [37]. In the present study, the Cronbach’s α for the emotional exhaustion, depersonalization and personal accomplishment dimensions was 0.86, 0.83, and 0.89, respectively (Additional file 2: Appendix 2).

##### Sense of coherence scale

The SOC scale was developed by Antonovsky to assess how people manage stressful situations and stay well [38].The Chinese version of the scale was introduced and revised by Bao Leiping [39].The scale includes 13 items, each of which is rated on a Likert scale ranging from 1 (“very common”) to 7 (“very rare or never”), including 3 dimensions: comprehensibility (5 items), manageability (4 items) and meaningfulness (4 items). The total scores range from 13 to 91, with higher scores indicating a stronger SOC. The Cronbach’s α of the scale was 0.76 [40]. In the present study, Cronbach’s α was 0.91 (Additional file 2: Appendix 2).

### Statistical analysis

Data were entered into and analysed using SPSS 26.0. First, we checked for missing values, outliers, and normality and excluded 85 missing data points before data analysis [41]. Second, summative score values for each scale were calculated, and the total scores of SOC, workplace violence and burnout were approximately normally distributed [41]. T tests and ANOVA were used to assess differences between the groups with regard to participant characteristics and selected variables [41]. The relationships between variables were examined via Pearson correlation analysis [41]. The PROCESS macro for SPSS was used to analyse the hypothesized mediation model, the approach based on ordinary least-squares regression and the bootstrap method [42]. Bootstrapping does not require the assumption of normality of the sampling distribution, and it has higher power while maintaining reasonable control over the Type I error rate [43]. Non-standardized beta coefficients are calculated to reduce Type 1 errors due to distribution. Pairwise contrasts are used to assess whether the three specific indirect effects are significantly different from one another comparisons if the 95% bias-corrected and accelerated confidence interval (CI) do not cross zero [43]. A previous study noted that Hayes recommended that 10,000 bootstrap samples be used for mediation analyses in the test from mediation Model 6 [42]. We used 10,000 bootstrap resamples to calculate the 95% CI, if the interval did not include zero, the effect was statistically significant at *p* < 0.05 [41]. Harman’s single-factor method was used to test for common method bias [44].

## Results

### Characteristics of participants and differences in workplace violence, sense of coherence and burnout

The explanatory power of variance for the largest factor was 30.61%, lower than 40%, indicating no significant common method bias in this study [44]. In addition, histograms and normal Q-Q plots of workplace violence, sense of coherence and burnout showed that the data were approximately normal (Supplementary Figs. 1, 2, 3 and 4). The baseline characteristics of the nurses are presented in Table 1. A total of 1105 valid questionnaires were collected. The participants included 57 (5.16%) males and 1048 (94.84%) females, and their average age was 32.92 ± 6.92 years. A total of 464 (41.99%) nurses experienced workplace violence. In addition, 41.00% (453/1105) and 4.80% (53/1105) of nurses experienced psychological violence and physical violence, respectively. Table 1 shows that nurses with different marital statuses (*F* = 4.302, *P* = 0.014), numbers of children (*F* = 5.462, *P* = 0.004), professional titles (*F* = 3.212, *P* = 0.041), departments (*F* = 7.012, *P* = 0.000), lengths of service (F = 3.019, *P* = 0.017) and positions (t = 3.202, *P* = 0.001) had significant differences in burnout scores. The results showed that the “other” type of marital status was associated with a higher level of burnout. Similarly, higher levels of burnout were found in nurses with lower professional title, fewer children, shorter lengths of service, and lower positions. Nurses who worked in surgery medicine had higher burnout. There were significant differences in the scores of workplace violence by professional title (*F* = 5.192, *P* = 0.006**),** department (*F* = 35.129, *P* = 0.000) and length of service (*F* = 3.323, *P* = 0.010).

**Table 1:** Participant characteristics and differences in workplace violence, sense of coherence and burnout [M(SD)]

Variable	N (%)	Workplace violence	*t / F*	*P*	sense of coherence	*t / F*	*P*	burnout	*t / F*	*P*
**Gender**										
Male	57	1.71 (0.98)	***t*** = -0.442	0.659	68.32 (12.18)	***t*** = -0.914	0.361	49.89 (17.19)	***t*** = 1.070	0.285
Female	1048	1.67 (1.08)			65.47 (13.29)			47.22 (18.46)		
**Age**										
≤ 30	462	1.56 (0.94)	***F*** = 2.149	0.092	66.26 (13.27)	***F*** = 2.679	0.046*	47.87 (18.73)	***F*** = 2.173	0.090
30–40	503	1.76 (1.16)			64.21 (13.32)			47.91 (18.31)		
41–50	124	1.70 (1.29)			66.40 (12.81)			43.85 (17.26)		
≥ 51	16	1.54 (0.88)			69.13 (10.07)			42.38 (17.60)		
**Educational level**										
Below the undergraduate	153	1.51 (0.99)	***F*** = 0.225	0.798	65.27 (13.38)	***F*** = 2.590	0.075	48.00 (20.34)	***F*** = 2.523	0.081
Undergraduate	929	1.70 (1.09)			65.56 (13.25)			47.05 (18.02)		
Master and above	23	1.81 (1.09)			59.22 (10.54)			55.57 (18.46)		
**Marital status**										
Single	256	1.56 (1.01)	***F*** = 0.651	0.522	63.33 (13.78)	***F*** = 5.914	0.003*	49.97 (19.74)	***F*** = 4.302	0.014*
Married	830	1.70 (1.09)			63.12 (13.07)			46.44 (17.85)		
Other	19	1.84 (1.42)			59.58 (8.66)			52.21 (19.96)		
**Only child**										
Yes	413	1.79 (1.19)	***t*** = 1.668	0.096	66.54 (13.39)	***t*** = 2.254	0.024*	46.85 (18.79)	***t*** = -0.709	0.478
No	692	1.60 (1.01)			64.69 (13.11)			47.66 (18.66)		
**Birth child**										
0	322	1.64 (1.06)	***F*** = 0.031	0.969	64.00 (13. 46)	***F*** = 3.395	0.034*	49.74 (19.11)	***F*** = 5.462	0.004*
1	556	1.68 (1.08)			65.55 (13.46)			47.13 (18.23)		
2	227	1.71 (1.09)			66.94 (12.17)			44.53 (17.36)		
**Professional titles**										
Primary title	599	1.55 (0.94)	***F*** = 5.192	0.006*	65.50 (13.44)	***F*** = 0.766	0.465	48.47 (18.89)	***F*** = 3.212	0.041*
Intermediate title	437	1.78 (1.20)			64.88 (12.96)			46.44 (17.72)		
Senior title	69	1.91 (1.48)			66.75 (13.24)			43.45 (17.59)		
**Department**										
Internal Medicine	296	1.58 (1.09)	***F*** = 35.129	0.000**	64.33 (13.58)	***F*** = 1.037	0.375	48.90 (18.76)	***F*** = 7.012	0.000**
Surgery Medicine	317	1.72 (1.12)			65.52 (12.94)			49.15 (17.51)		
Outpatient and Emergency	179	2.13 (2.02)			65.45 (13.24)			48.61 (18.85)		
Other	313	1.04 (0.49)			66.21 (13.20)			43.36 (18.13)		
**Length of service**										
≤ 1 year	36	0.93 (0.39)	***F*** = 3.323	0.010*	67.00 (10.86)	***F*** = 0.767	0.547	47.56 (16.65)	***F*** = 3.019	0.017*
2–5 years	201	1.68 (1.12)			65.10 (13.33)			48.89 (19.38)		
6-10 years	346	1.61 (0.93)			65.82 (13.84)			48.46 (19.48)		
11-15 years	268	1.75 (1.13)			64.34 (13.38)			48.03 (17.19)		
≥ 16 years	254	1.75 (1.28)			65.89 (12.47)			43.90 (17.20)		
**Position**										
Common nurse	1016	1.67 (1.05)	***t*** = -1.957	0.051	65.26 (13.22)	***t*** = -1.007	0.314	47.88 (18.48)	***t*** = 3.202	0.001*
Head nurse	87	1.76 (1.41)			66.75 (13.56)			41.32 (16.51)		

Note: * P < 0.05; **P < 0.001

### Descriptive and correlation analysis of workplace violence, sense of coherence and burnout

Table 2 shows the Pearson correlations (two-tailed) for workplace violence, SOC and burnout. The mean total scores for workplace violence were 1.67 ± 1.08 (range = 0–27), and the scores for SOC and burnout were 65.39 ± 13.24 (range = 13–91) and 47.36 ± 18.39 (range = 0–132), respectively. Pearson’s correlations showed that workplace violence was significantly negatively correlated with SOC (*r* = -0.247, *P* < 0.001) and positively correlated with burnout (*r* = 0.273, *p* < 0.001). Additionally, SOC was significantly negatively correlated with burnout (*r* = -0.731, *P* < 0.001).

**Table 2:** Descriptive statistics and correlation of workplace violence, sense of coherence and burnout (*N* = 1105)

Item	Range	M ± SD	95%CI	1	2	3	4	5	6	7	8	9
1 Workplace violence	0–27	1.67 ± 1.08	0.98–1.18	1								
2 Sense of coherence	13–91	65.39 ± 13.24	64.60–66.17	-0.247**	1							
3 Comprehensibility	5–35	25.03 ± 5.54	24.70–25.35	-0.221**	0.941**	1						
4 Manageability	4–28	20.45 ± 4.47	20.19–20.71	-0.262**	0.923**	0.823**	1					
5 Meaningfulness	4–28	19.91 ± 4.41	19.65–20.17	-0.198**	0.884**	0.733**	0.723**	1				
6 Burnout	0–132	47.36 ± 18.39	46.27–48.44	0.273**	-0.731**	-0.650**	-0.656**	-0.714**	1			
7 Emotional exhaustion	0–54	20.59 ± 8.69	20.08–21.11	0.285**	-0.615**	-0.566**	-0.579**	-0.546**	0.834**	1		
8 Depersonalization	0–30	6.39 ± 4.57	6.12–6.66	0.293**	-0.601**	-0.522**	-0.549**	-0.592**	0.799**	0.632**	1	
9 Personal accomplishment	0–48	20.38 ± 9.15	19.84–20.92	0.133**	-0.587**	-0.509**	-0.495**	-0.620**	0.819**	0.413**	0.508**	1

*Note:* **P < 0.001

*CI* Confidence Interval

### Mediating role of sense of coherence between workplace violence and burnout

**Fig. 2:**
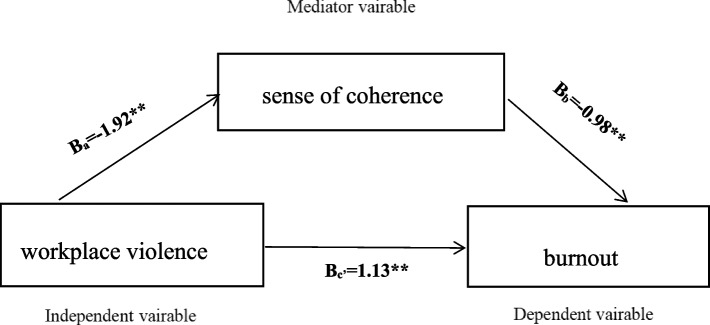
Mediating model of workplace violence, sense of coherence and burnout. Note: **p < 0.001

Table 3 shows that all individual paths between the key variables in the model were significant after accounting for covariates. The R^2^ and adjusted R^2^ of the model were 0.5603 and 0.5580, respectively. The model would be counted as a good fit. In addition, the residual plot of the model showed the fitness of the model was good (Supplementary Fig. 5). Higher levels of workplace violence were associated with higher burnout (*β* = 1.1293, 95% CI: 0.6790 ~ 1.5797). Higher SOC was associated with lower burnout (*β* = -0.9772, 95% CI: -1.0339 ~ -0.9205), as shown in Fig. 2. Additionally, Table 4 shows that the total effect (effect = 3.0079, SE = 0.3181, t = 9.5461, *p* < 0.001) and the direct effect (effect = 1.1293, SE = 0.2295, t = 4.9205, *p* < 0.001) of workplace violence on burnout were found to be significant. The indirect effect was 1.8785 (95% CI: 1.3871 ~ 2.4242), which accounted for 62.45% of the total effect (3.0079). The model was not at a zero-point estimate interval within the 95% CI. Therefore, the indirect effect was statistically significant. SOC plays a mediating role between workplace violence and burnout among Chinese nurses.

**Table 3:** Results of the regression analyses testing the mediating effect of sense of coherence in the relationship workplace violence and burnout (*N* = 1105)

Predictors	Beta	SE	t	*p*	95%CI	
					**LLCI**	**ULCI**
Constant	118.7002	2.7745	42.7833	0.0000**	113.2563	124.1440
Sense of coherence	-0.9772	0.0289	-33.7976	0.0000**	-1.0339	-0.9205
Workplace violence	1.1293	0.2295	4.9205	0.0000**	0.6790	1.5797
Position	-3.5007	1.5573	-2.2480	0.0248*	-6.5562	-0.4451
Department	-0.9772	0.3209	-3.0452	0.0024*	-1.6068	-0.3475
Length of service	-1.3265	0.5776	-2.3007	0.0216*	-2.4579	-0.1952

Note: * *P* < 0.05; **P < 0.001, Unstandardized regression coefficients (Beta) with standard error (SE) in parentheses are presented

*CI* Confidence Interval

**Table 4:** Bootstrapped point estimates with standard errors and 95% confidence intervals for all indirect effects between workplace violence and burnout (*N* = 1105)

Items	Beta /Effect	SE	t	*P*	95% CI	
					**LLCI**	**ULCI**
**Path way**						
workplace violence → burnout	3.0079	0.3181	9.4561	0.0000**	2.3837	3.6320
workplace violence → sense of coherence	-1.9223	0.2325	-8.2669	0.0000**	-2.3786	-1.4661
sense of coherence → burnout	-0.9772	0.0289	-33.7976	0.0000**	-1.0339	-0.9205
**Effects**						
Direct effect	1.1293	0.2295	4.9205	0.0000**	0.6790	1.5797
Indirect effect^a^	1.8785	0.2606			1.3871	2.4242
Total effect	3.0079	0.3181	9.4561	0.0000**	2.3837	3.6320

^a^Based on 10000 bootstrap samples

*CI* Confidence Interval

## Discussion

There are three major findings in our study. (1) During the epidemic, the prevalence of workplace violence was high among Chinese nurses. It was negatively correlated with SOC and positively correlated with burnout. (2) A higher level of workplace violence was associated with higher burnout, while a higher SOC was associated with lower burnout. (3) SOC plays a mediating role between workplace violence and burnout among Chinese nurses. These findings suggest that managers of the health care workforce should be aware of the risks of workplace violence among nurses as well as the consequent psychological distress (burnout) and should provide better training and support for nurses from multidimensional and positive perspectives on SOC to adapt to related stressors.

### Descriptive analysis and social characteristics of participants for workplace violence, SOC and burnout

Our research shows that the prevalence of workplace violence among nurses (41.99%) is relatively lower than a previous result (68.31%) [45]. The prevalence of psychological violence and physical violence was 41.00% (453/1105) and 4.80% (53/1105), respectively, lower than Wu’s results (77.6% and 35.1%, respectively) [6] and Li’s results (67.28% and 37.63%, respectively) [46].This difference may be related to the introduction of new policies by the Chinese government to increase penalties for workplace violence. Another important reason is that nurses play an irreplaceable role in the prevention and control of the epidemic, and the majority of Chinese people are grateful to them. However, the prevalence of workplace violence is still high because some patients’ health needs are not met in a timely manner, which makes nurses easily become the main victims of workplace violence in China. In addition, this study found that the type of violence was mainly psychological violence, consistent with previous research results [47, 48]. Psychological violence is the basis of physical violence. When a patient has negative emotions in the process of medical treatment, he or she will first choose verbal abuse to express his or her dissatisfaction [49]. Therefore, the incidence of psychological violence was high. In addition, our results revealed that nurses’ professional titles, department and length of service were common influencing factors of burnout and workplace violence. The results were similar to a previous study [50]. Currently, most nurses in China are contract workers; they have poor job stability, and they must pay more for their work to keep their jobs. In certain periods, due to their sense of mission and responsibility, they invest more at work and hope to achieve higher levels of recognition and respect. Therefore, when effort-reward imbalance occurs (especially more effort, less reward with regard to respect), individuals will have higher pressure and burnout [51].

### Correlation between workplace violence, SOC and burnout

Our research showed that workplace violence was positively correlated with burnout and that nurses with higher levels of workplace violence were associated with higher burnout, similar to the findings of previous studies [46, 52, 53]. Public opinion suggests that patients are vulnerable, lack medical knowledge and are affected by a “consumer psychology”. The information exchange between nurses and patients or their families is inefficient because of the lack of medical human resources, which makes it difficult to meet patients’ psychological expectations. This situation may promote the development of workplace violence. Workplace violence can lead to nurses’ anxiety, fear and fatigue as well as depression. This can affect nurses’ self-confidence in their work and lead to ineffective coping with workplace violence and depersonalization [54]. Workplace violence also leads to nurses’ excessive cognitive processing for adverse stress and the cognitive bias of intrusive thoughts [54]. Intrusive thoughts caused by threatening stressors are an abnormal cognitive processing of repeated meaningless stress events [55]. When individuals have intrusive thoughts, they correct them through self-control to meet social requirements (with patient-centred concepts, nurses are required to consider the emotions of patients and their families) and self-standards. The process of self-control leads to the depletion of psychological resources [54]. These lost psychological resources require a long period of time to recover, and people’s self-regulation ability declines when they lack psychological resources [56, 57]. People may be negative due to a lack of psychological resources, and a lack of effective coping strategies to cope with workplace violence further aggravates depersonalization. Moreover, because of negative emotional behaviour feedback, nurses lack work confidence and show low personal accomplishment, forming a "domino effect" that aggravates nurses’ burnout [58]. Moreover, affective event theory suggests that positive or negative affective work events could lead to affective experiences, subsequently influencing work attitudes [59]. Violence not only threatens the physical safety of nurses but also causes emotional frustration and reduces work enthusiasm; it may especially lead to nurses’ emotional exhaustion [36]. One study showed that compared with doctors, nurses suffered from more workplace violence [11]. Although nurses play a very important role in the prevention and control of epidemics, the traditional view of doctors ignoring nursing remains difficult to change in China. Nurses who have experienced violence face a crisis of professional honour due to a lack of sufficient social respect and understanding, which leads to a negative evaluation of nurses’ professional pride and makes nurses’ burnout more severe.

### Mediating role of SOC on workplace violence and burnout

This study sought to explain the mediating role of SOC between workplace violence and burnout among Chinese nurses based on the stress system model. Our study showed that workplace violence not only has direct effects on burnout but also has indirect effects on burnout via SOC. The results are similar to those of previous studies [24, 26] that showed that SOC is a partial mediator of the relationships between work-related violence and various stress reactions [26]. According to transactional models of stress [60, 61], the outcome of a stressful transaction is mediated by the individual’s appraisal and coping. Exposure to violence at work is a potent psychosocial stressor, and SOC plays an important role in coping with stressors [26]. Nurses with high-level SOC possess a greater capacity to face stressful situations and mobilize available resources, both their own and those of their workplace [62]. This could have repercussions in terms of greater dedication, greater vigour and, ultimately, less burnout. A previous study showed that the SOC can affect medical professionals’ burnout, and the influence of the “meaningfulness” dimension is most significant [63]. When medical professional**s** feel their work is meaningless or when they feel they have no confidence or do not want to invest energy and responsibility to deal with stressors at work, burnout will appear or increase [63]. Similarly, another study showed that SOC can prevent burnout and that “manageability” and “meaningfulness” can alleviate burnout [25]. Consequently, individual differences in how nurses perceive and cope with workplace violence may partly explain why some nurses develop severe burnout following exposure to workplace violence while others seem to be relatively unaffected. The pressure produced by work does not directly lead to nurses’ burnout but is caused by the imbalance in the adjustment of nurses’ internal resources like resilience [19, 64]. SOC can be used as effective psychological capital to help nurses cope with burnout from work.

There are some limitations in this study. First, this was a cross-sectional study. It will be necessary to design a longitudinal study to test the dynamic relationship. Second, the proportion of male nurses in the study was low. It is difficult to reflect gender differences in workplace violence. Future research should focus on the occurrence of male nurses in workplace violence by randomized sampling. Third, workplace violence from internal organizations was not included in the study. Future research should include internal organizations. Fourth, many individuals may seek psychotherapeutic support, or in some cases, counseling at multiple levels during the period. This information was unavailable, it could influence the characteristics of the respondents to the questionnaire. Despite its limits, the present research also has strengths. A major strength is that we found that the prevalence of workplace violence was still high among Chinese nurses during the COVID-19 pandemic. The literature highlights the important role of SOC in the relationship between workplace violence and burnout among Chinese nurses. In addition to conventional psychological intervention strategies to reduce burnout caused by workplace violence, nursing managers should pay more attention to the SOC of nurses. A study has shown that nurses’ SOC levels can increase with increasing cognitive behaviour [65]. Therefore, nursing managers can adopt cognitive behavioural interventions based on psychological rewards to improve nurses’ SOC to mitigate the negative impact of workplace violence [66]. In addition, SOC is an internal motivating factor for the development of individual mental health and a lasting and stable internal force. Nursing educators should integrate SOC in the teaching process in a coherent and understandable way to encourage nursing students to establish internal psychological capital so that they can better cope with complex challenges in their future work and life. For policy-makers, a series of care and supervision mechanisms should be established to ensure better implementation of measures to promote nurses’ mental health.

## Conclusions

The results from the present study support the hypothesis. We found that SOC played a mediating role between workplace violence and burnout among Chinese nurses. Furthermore, workplace violence negatively affected SOC and positively affected burnout in nurses, whereas SOC played a protective role between them. Workplace violence is an inevitable occurrence. The research demonstrated preliminary relationship mechanisms for workplace violence, SOC and burnout in nurses. The results provide a reference for hospital management and intervention program designers to reduce the occurrence of burnout by enhancing nurses’ SOC.

### Supplementary Information

**Additional file 1.** STROBE Statement—Checklist of items that should be included in reports of cross-sectional studies.

**Additional file 2.** Measurement Questionnaire.

**Additional file 3: Supplementary Figure 1.** The normal histogram of burnout.

**Additional file 4: Supplementary Figure 2.** The normal Q-Q chart of burnout.

**Additional file 5: Supplementary Figure 3.** The normal QQ chart of workplace violence.

**Additional file 6: Supplementary Figure 4.** The normal histogram of sense of coherence.

**Additional file 7: Supplementary Figure 5.** The residual plot of the model.

## Data Availability

The data sets used and /or analysed during the current study are available from the corresponding author on reasonable request. The data are not publicly available due to privacy or ethical restrictions.

## Abbreviations

SOC: Sense of coherence
CI: Confidence interval
