# Supplementary material for: Workplace violence and burnout among Chinese nurses during the COVID-19 pandemic: does the sense of coherence mediate the relationship?
Source: BMC Psychiatry. 2023 Aug 8;23:573. doi: 10.1186/s12888-023-05060-9 (PMC10408152; doi:10.1186/s12888-023-05060-9)
Supplement: Supplementary file 2 — Additional file 2. Measurement Questionnaire. [file 12888_2023_5060_MOESM2_ESM.pdf]

Questionnaires

Demographic characteristics questionnaire

1. Your gender： ①Male； ②Female

2. Your age： ______years；

3. Your educational level：①Below the undergraduate； ②Undergraduate； ③Master and above

4. Your position：①Common nurse； ②Head nurse

5. Your professional titles：①Primary title； ②Intermediate title； ③Senior title

6. Your marital status：①Single； ②Married； ③Other

7. Your birth child：①0； ② 1； ③ 2

8. Your length of service：①≤1 year ②2-5 years ③6-10years ④11-15years ⑤≥16years

9. Are you only child? ①Yes ②No

10. Your department：①Internal Medicine ②Surgery Medicine ③Outpatient and Emergency ④Other

Workplace Violence Scale

Introduction: The following questions are about your work experience in the past 12 years, which was caused by the patient/ patient's family member. Each question has a choice of items. Please read each option carefully and then choose the option that best expresses your feelings, thank you for your cooperation!

1.In the past 12 months, have you been scolded, verbally abused, abused, belittled or otherwise demeaning to your dignity in the hospital (in person, by phone, by letter or leaflet, via WeChat, etc.)?。

① never ②once ③twice ④three or more times

2. In the past 12 months, has anyone made threats against you in the hospital: threats regarding personal and property safety, threatening complaints, stalking, etc?

① never ②once ③twice ④three or more times

3. In the past 12 months, has anyone physically assaulted you in the hospital, causing no physical injury?

① never ②once ③twice ④three or more times

4.In the past 12 months, has anyone caused you a minor injury in the hospital, such as pain, bruising, scratching or bruising?

① never ②once ③twice ④three or more times

5. In the past 12 months, has anyone done anything to you in the hospital that has caused a visible injury, such as a wound, fracture, internal organs or head injury?

① never ②once ③twice ④three or more times

6. In the past 12 months, has anyone done anything to you in the hospital that has caused serious consequences such as dysfunction or permanent disability?

① never ②once ③twice ④three or more times

7. In the past 12 months, have you been sexually harassed or made sexual advances at the hospital, including words, movements, or the exposure of sexual organs?

① never ②once ③twice ④three or more times

8. In the past 12 months, have you been sexually assaulted in the hospital, such as being pulled, held, kissed, or touched by someone of the opposite sex?

① never ②once ③twice ④three or more times

9. In the past 12 months, has anyone raped or attempted to rape you in the hospital?

①never ②once ③twice ④three or more times

Burnout Inventory

Introduction: Please read each item carefully and then according to the frequency of occurrence in the last month and then choose the option. Requirement: An independent self-assessment that is not influenced by anyone.

MBI Human Services Survey

How often:	0	1	2	3	4	5	6
	Never	A few times a year or less	Once a month or less	A few times a month	Once a week	A few times a week	Every day

How often 0-6	Statements:
1. ________	I feel emotionally drained from my work.
2. ________	I feel used up at the end of the workday.
3. ________	I feel fatigued when I get up in the morning and have to face another day on the job.
4. ________	I can easily understand how my recipients feel about things.
5. ________	I feel I treat some recipients as if they were impersonal objects.
6. ________	Working with people all day is really a strain for me.
7. ________	I deal very effectively with the problems of my recipients.
8. ________	I feel burned out from my work.
9. ________	I feel I'm positively influencing other people's lives through my work.
10. ________	I've become more callous toward people since I took this job.
11. ________	I worry that this job is hardening me emotionally.
12. ________	I feel very energetic.
13. ________	I feel frustrated by my job.
14. ________	I feel I'm working too hard on my job.
15. ________	I don't really care what happens to some recipients.
16. ________	Working with people directly puts too much stress on me.
17. ________	I can easily create a relaxed atmosphere with my recipients.
18. ________	I feel exhilarated after working closely with my recipients.
19. ________	I have accomplished many worthwhile things in this job.
20. ________	I feel like I'm at the end of my rope.
21. ________	In my work, I deal with emotional problems very calmly.
22. ________	I feel recipients blame me for some of their problems.

Sense of Coherence scale

Introduction: Here are questions about all aspects of your work. Each question has 7 items to choose from. Please read each option carefully and then choose the option that best expresses your feelings. Thank you for your cooperation!

1. Do you often feel like you don't care about what's going on around you?

①Very rare or never ②Seldom ③Less ④Hard to say ⑤Sometimes ⑥More ⑦Very common

2. Did it happen a lot in the past that someone you thought you knew well would surprise you?

①Very rare or never ②Seldom ③Less ④Hard to say ⑤Sometimes ⑥More ⑦Very common

3. You're counting on someone to let you down.

①Very rare or never ②Seldom ③Less ④Hard to say ⑤Sometimes ⑥More ⑦

Very common

4. Do you often feel that you are being treated unfairly?

①Always feel ②Have this feeling ③I feel like that once in a while ④Hard to say ⑤Seldom ⑥Hardly ⑦Never have

5. Do you often find yourself in unfamiliar situations where you don't know what to do?

①Very common ②More③Sometimes ④Hard to say ⑤Less ⑥Seldom ⑦Very rare or never

6. Do you often have very complex, mixed feelings and thoughts?

①Very common ②More③Sometimes ④Hard to say ⑤Less ⑥Seldom ⑦Very rare or never

7. Do you often have emotions you don't want to have?

①Very common ②More③Sometimes ④Hard to say ⑤Less ⑥Seldom ⑦Very rare or never

8. Many people, even very talented people, sometimes feel like failures under certain circumstances. Have you often had this feeling of failure in the past?

①Very rare or never ②Seldom ③Less ④Hard to say ⑤Sometimes ⑥More ⑦ Very common

9. How often do you think that what you do every day is meaningless?①Very common ②More③Sometimes ④Hard to say ⑤Less ⑥Seldom ⑦Very rare or never

10. Do you often feel out of control?

①Very common ②More③Sometimes ④Hard to say ⑤Less ⑥Seldom ⑦Very rare or never

11. Your life so far:

①There is no goal at all.②Have no goal ③Not very purposeful④Hard to say ⑤ Have a goal in life

⑥The goal of life is more clear ⑦The goal of life is very clear

12. When faced with problems or things, you find yourself generally_____

①Underestimate or overestimate its importance ②Hard to gauge ③A little off estimate

④Hard to grasp⑤Evaluate the matter more accurately⑥Evaluate the matter accurately ⑦Appraise quite correctly

13. Do what you do every day for you ______

①It's great joy and satisfaction ②is more happy and satisfied ③is a little happy ④ is hard to say ⑤ is a little unhappy ⑥is very easy to steal ⑦is a source of pain and annoyance
