# Supplementary figures and images for: Workplace violence and burnout among Chinese nurses during the COVID-19 pandemic: does the sense of coherence mediate the relationship?
Source: BMC Psychiatry. 2023 Aug 8;23:573. doi: 10.1186/s12888-023-05060-9 (PMC10408152; doi:10.1186/s12888-023-05060-9)

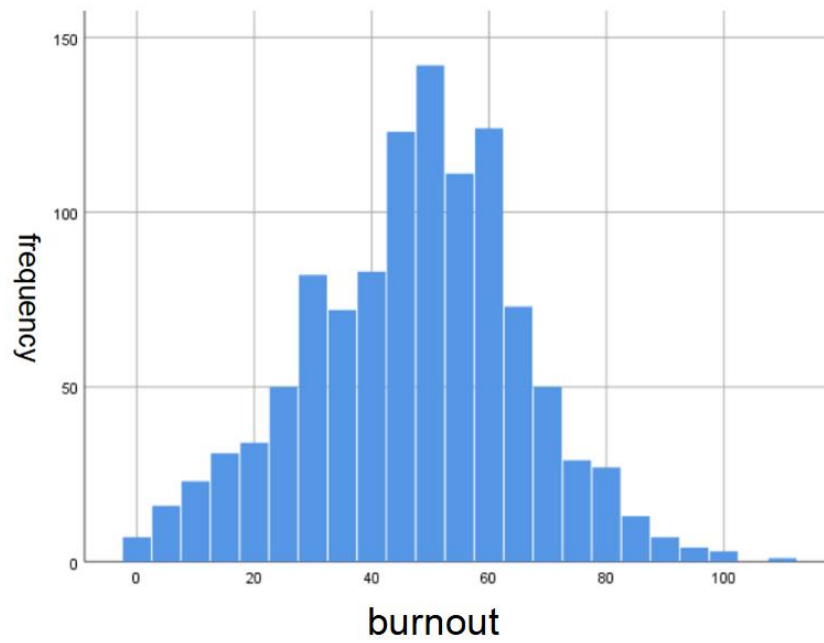

**Supplementary figure 1. The normal histogram of burnout**

Supplement: Supplementary file 3 — Additional file 3: Supplementary Figure 1. The normal histogram of burnout. [file 12888_2023_5060_MOESM3_ESM.pdf]

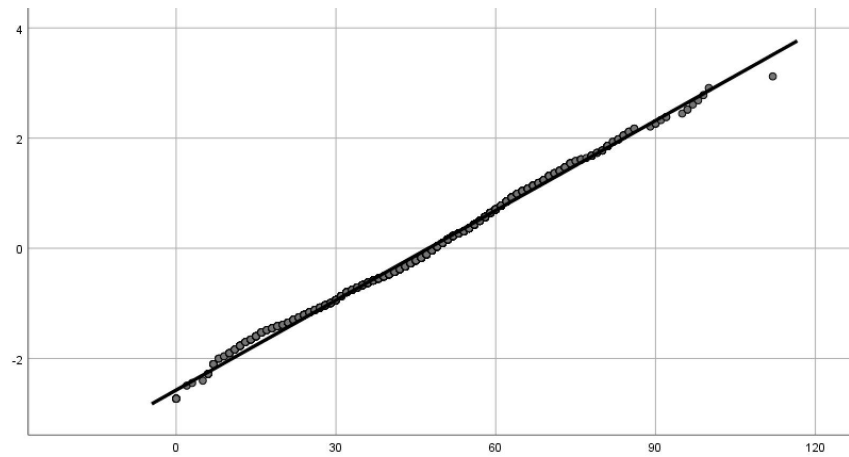

**Supplementary figure 2. The normal Q-Q chart of burnout**

Supplement: Supplementary file 4 — Additional file 4: Supplementary Figure 2. The normal Q-Q chart of burnout. [file 12888_2023_5060_MOESM4_ESM.pdf]

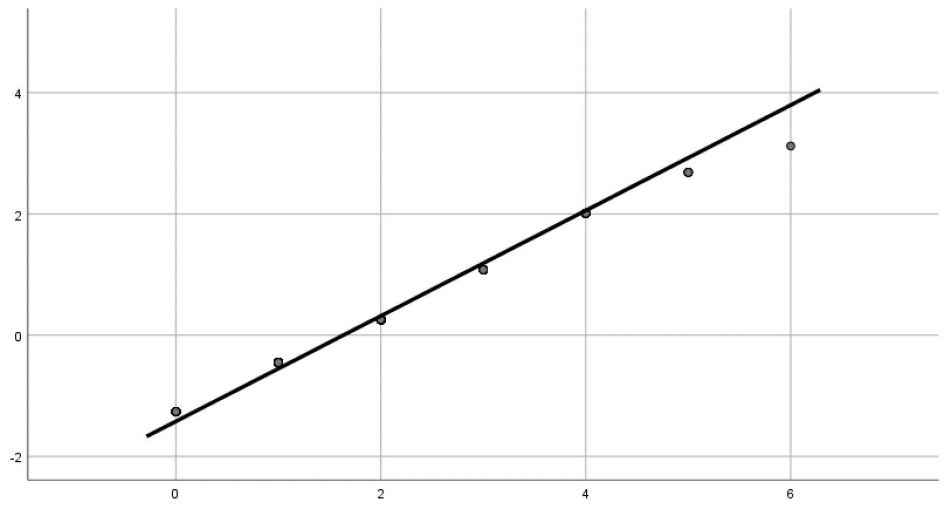

**Supplementary figure 3. The normal QQ chart of workplace violence**

Supplement: Supplementary file 5 — Additional file 5: Supplementary Figure 3. The normal QQ chart of workplace violence. [file 12888_2023_5060_MOESM5_ESM.pdf]

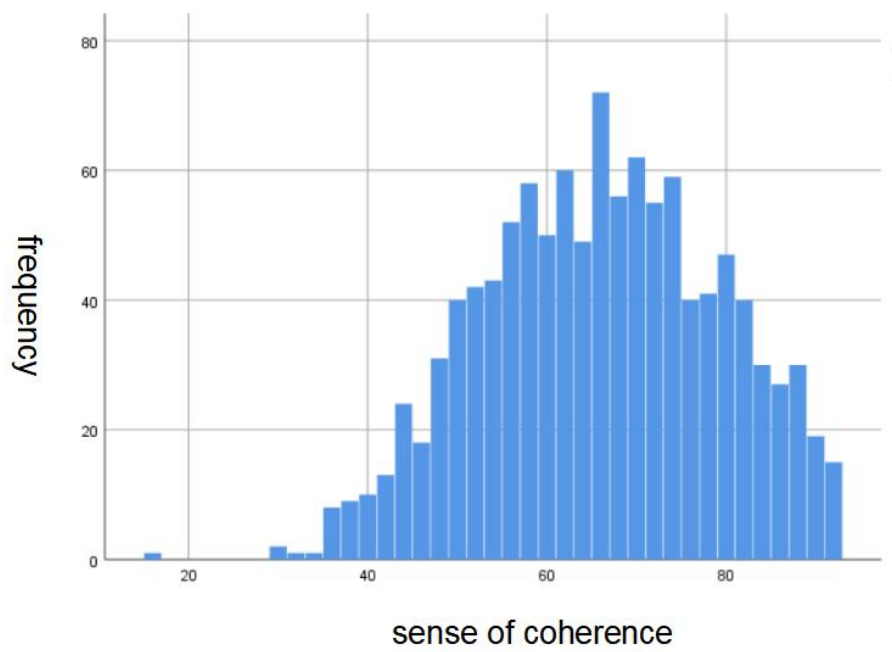

**Supplementary figure 4. The normal histogram of sense of coherence**

Supplement: Supplementary file 6 — Additional file 6: Supplementary Figure 4. The normal histogram of sense of coherence. [file 12888_2023_5060_MOESM6_ESM.pdf]

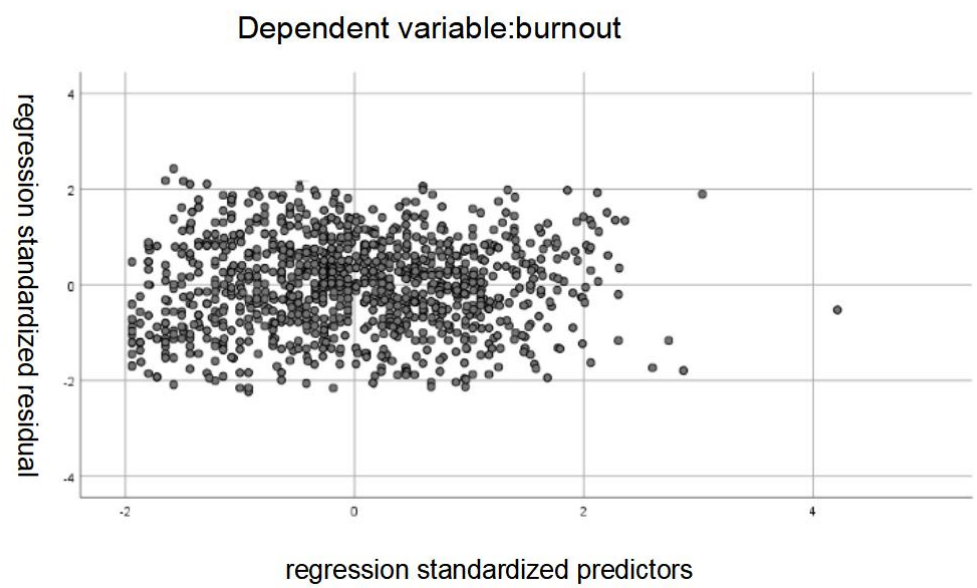

**Supplementary figure 5. The residual plot of the model**

Supplement: Supplementary file 7 — Additional file 7: Supplementary Figure 5. The residual plot of the model. [file 12888_2023_5060_MOESM7_ESM.pdf]
